# Association of carotid artery stenosis with cerebral artery signal intensity gradient on time-of-flight magnetic resonance angiography

**DOI:** 10.3389/fneur.2025.1576655

**Published:** 2025-08-11

**Authors:** Chan-Hyuk Lee, Seul-Ki Jeong, Hyun Jin Kim, Robert S. Rosenson, Wookjin Yang, Keun-Hwa Jung

**Affiliations:** ^1^Department of Neurology, Ulsan University Hospital, University of Ulsan College of Medicine, Ulsan, Republic of Korea; ^2^Institute for Blood Flow and Metabolism Research, MediIMG, Inc., Seoul National University Bundang Hospital, Seongnam, Republic of Korea; ^3^Korea Advanced Institute of Science and Technology, Mechanical Engineering, Daejeon, Republic of Korea; ^4^Fuster Heart Hospital, Icahn School of Medicine at Mount Sinai, New York, NY, United States; ^5^Department of Neurology, Asan Medical Center, Seoul, Republic of Korea; ^6^Department of Neurology, Seoul National University Hospital, Seoul National University College of Medicine, Seoul, Republic of Korea

**Keywords:** basilar artery, internal carotid artery stenosis, signal intensity gradient, time-of-flight magnetic resonance angiography, wall shear rate

## Abstract

**Purpose:**

Extracranial internal carotid artery (ICA) stenosis is a known cause of large artery ischemic stroke. However, its association with cerebral arterial hemodynamics has been relatively underexplored. This study investigates the relationship between extracranial ICA stenosis and signal intensity gradient (SIG) in major cerebral arteries. The SIG is a surrogate marker for arterial wall shear rate.

**Methods:**

In the cross-sectional, retrospective study, we included individuals who underwent health screenings for vascular risk factors, as well as Time-of-Flight Magnetic Resonance Angiography and carotid Doppler ultrasonography. Extracranial ICA stenosis was categorized into three groups: normal, <50% stenosis, and ≥50% stenosis. In each group, SIGs were measured in major cerebral arteries. The association between ICA status and SIG in major cerebral arteries was analyzed using logistic regression, with SIG tertiles as the variable of interest.

**Results:**

A total of 1,138 individuals (mean age ± SD, 63.3 ± 9.6 years) were included. ICA stenosis ≥50% was significantly associated with cerebral artery SIGs, with age and basilar artery (BA) SIG showing the strongest correlations. Multinomial logistic regression revealed that individuals in the lowest tertile of BA SIG had a significantly higher odds ratio (OR) for ICA stenosis (OR: 2.72, 95% CI: 1.27–5.82; *p* = 0.010) compared to those in the highest tertile.

**Conclusion:**

ICA stenosis is significantly correlated with BA SIG, indicating a possibility of link between ICA stenosis and intracranial hemodynamics. A prospective longitudinal study is warranted to clarify the causal link between ICA stenosis and BA SIG.

## 1 Introduction

Atherothrombosis is a key pathological process in vascular diseases. Under conditions of laminar flow, shear rates are highest at the arterial wall, influencing endothelial cell morphology and function (1) as endothelial cells elongate and align with the direction of flow (2, 3). The secretion and release of endothelial protective factors, such as nitric oxide (NO) and tissue plasminogen activator (t-PA), are shear-dependent (4, 5). The upregulation of atherothrombotic and inflammatory endothelial mediators, encompassing endothelin, nuclear factor kappa-light-chain-enhancer of activated B cells (NF-κB), monocyte chemoattractant protein-1 (MCP-1), intercellular adhesion molecule 1 (ICAM-1) and vascular cell adhesion molecule 1 (VCAM-1) is also shear-dependent (6, 7).

The shear rate represents the rate of change in velocity between fluid layers relative to their distance from the centerline. Acting on the endothelium and can provide insights into the pathophysiological mechanisms of cerebrovascular diseases (CVD). To determine the shear rate along the arterial wall, phase contrast magnetic resonance (PC-MR), ultrasonography, and computational fluid dynamics (CFD) have been employed (8). PC-MR and ultrasonography estimate shear rate as 8 × velocity/diameter in arterial cross-sections based on the Hagen-Poiseuille equation, while CFD calculates shear rate position-sensitively as du/dr using the Navier–Stokes equation.

Three-dimensional (3D) time-of-flight magnetic resonance angiography (TOF-MRA) is one of the most widely used non-invasive techniques for assessing arterial luminal status. 3D TOF-MRA operates by capturing signals generated from the inflow of fresh, unsaturated, and fully magnetized blood spins into the imaging slab (9). Previous studies demonstrated that near-wall images from 3D TOF-MRA are well-preserved for deriving shear rates, a measure designated as the signal intensity gradient (SIG) (10). SIG derived from TOF-MRA has shown strong correlations with wall shear stress as measured by CFD and PC-MR (10, 11). Cerebral arterial SIG has been established as an image-based hemodynamic parameter in ischemic stroke (12, 13), rupture-prone aneurysms (14), and moyamoya disease (15).

Extracranial internal carotid artery (ICA) stenosis is recognized as a cause of large artery atherosclerosis (LAA) type ischemic stroke. Extracranial ICA stenosis has been extensively studied for its local geometric and hemodynamic characteristics (16), but its association with cerebral arterial hemodynamics has been less explored. In this study, we investigated the clinical correlates of major cerebral arterial SIG in relation to extracranial ICA stenosis in a population without prior cardio-cerebrovascular event.

## 2 Materials and methods

### 2.1 Research population

This cross-sectional and retrospective study included institutional individuals who visited a major healthcare center for health screenings between January 2015 and June 2021. Participants had no prior history of cardio-cerebrovascular disease before health screening. During the health check-up, participants underwent intracranial TOF-MRA and carotid Doppler ultrasonography (CDU). The inclusion criteria were: (1) age over 18 years; (2) no history of cardio-cerebrovascular disease before the index health check-up. To eliminate potential cerebrovascular event influences on hemodynamics, patients with previous stroke history were excluded. Additionally the following exclusion criteria were included: (1) inadequate intracranial TOF-MRA image quality due to motion artifacts, metal-induced distortions, or technical issues; (2) carotid stenosis from non-atherosclerotic causes such as vasculitis, radiation arteriopathy, congenital malformations, or fibromuscular dysplasia; and (3) history of revascularization or stenting of intra- or extracranial arteries. This study was approved by the Review Board (Seoul National University Hospital, No. H-2204-116-1317), and informed consent was waived due to its retrospective design.

### 2.2 Acquisition of clinical data

All patients were anonymized prior to data collection. Clinical data were collected from electronic medical records, including demographics, cardiovascular risk factors (hypertension, diabetes mellitus, hyperlipidemia, smoking history, intracranial artery stenosis (ICAS), atrial fibrillation (AF), and chronic kidney disease). Vital signs during health check-ups and laboratory results were included. AF was identified by history, treatment, or detection during health screenings. ICAS was diagnosed if stenosis was visible in the distal ICA or MCA (M1/M2) on MRA.

### 2.3 Parameters of intracranial time-of-flight magnetic resonance angiography

A 1.5 T or 3.0 T MRI scanner with a 32-channel head coil (Discovery MR750W, GE Healthcare, Chicago, IL; Magnetom Skyra, Siemens, Erlangen, Germany; Magnetom Verio, Siemens; IngeniaCX, Philips, Amsterdam, Netherlands) was used to acquire three-dimensional intracranial TOF images. The MRI parameters for the examination were as follows: slice number, 106–264; slice thickness, 0.47–1.2 mm; interslice gap, 0 mm; field of view, 165–220 × 210–240 mm; matrix size, 384–580 × 180–323 mm; repetition time (TR), 19–30 ms; echo time (TE), 3.4–7 ms; and flip angle (FA), 18–25°.

### 2.4 Assessment of SIG of cerebral arteries

The SIG of the major cerebral arteries, derived from the TOF-MRA in the Digital Imaging and Communications in Medicine (DICOM) format, was used as a surrogate marker for arterial wall shear rate. The cerebral arteries of measurement included bilateral ICA, MCA, anterior cerebral artery (ACA), posterior cerebral artery (PCA), and the basilar artery (BA). The assessment was performed using semi-automated software (VINT, MediIMG, Inc., Seoul, Republic of Korea) which has been approved by the U.S. Food and Drug Administration. Arterial wall SIG corresponds conceptually to shear rate (or velocity gradient) and has been validated as a marker for wall shear rate or stress through both CFD (10) and two-dimensional (2D) PC-MR imaging (11). The measurement and interpretation of SIG were anonymously performed by KH, who has more than 10 years of research experience in digital imaging.

**Figure 1:**
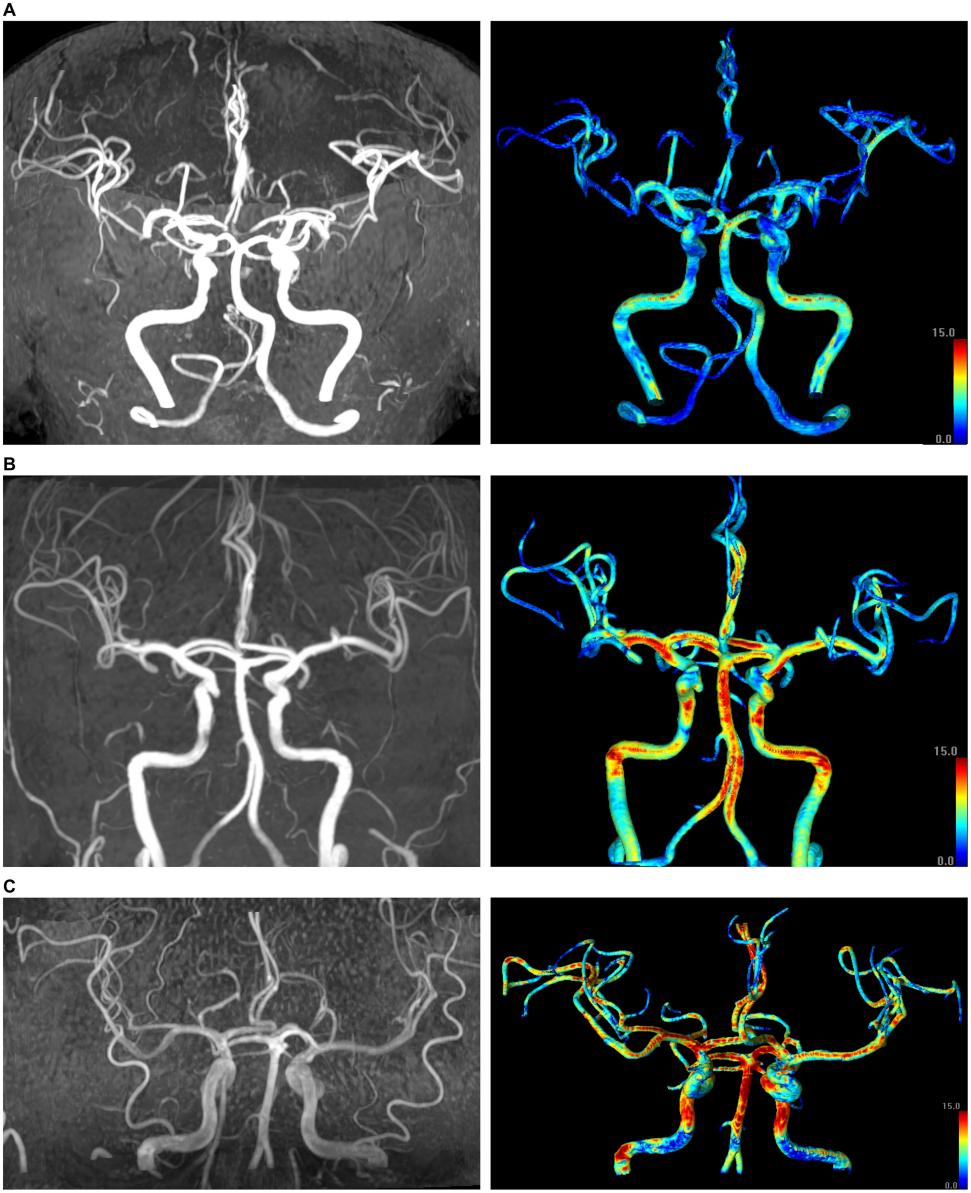
Representative cases in the three groups based on the mean signal intensity gradient (SIG) values of the basilar artery (BA). Left panel: maximum intensity projection images of time-of-flight magnetic resonance angiography (TOF-MRA); right panel: corresponding color display of SIG in the cerebral arteries. The color gradient indicates that red hues correspond to higher SIG values, while blue hues correspond to lower SIG values. **(A)** A 73-year-old female subject in the lowest tertile of BA SIG (mean BA SIG: 5.0 SI/mm). **(B)** A 77-year-old male subject in the second tertile (mean BA SIG: 9.6 SI/mm). **(C)** A 47-year-old male subject in the highest tertile (mean BA SIG: 12.9 SI/mm). All three images were acquired using identical MR parameters across different scanners: flip angle of 18°, repetition time of 20.0 ms, and echo time of 3.5 ms.

Arterial wall SIG was measured on the straightest segments to ensure laminar flow areas (Supplementary Figure 1). For the ICA, the C1 distal segment before the horizontal intrapetrous segment was selected. For the BA, the mid-to-distal segment was chosen. For the MCA and ACA, the proximal half or one-third was used. Lastly, the P1 segment was selected for the PCA. Representative cases illustrated maximum intensity projection images of TOF-MRA and three-dimensional reconstructions of arterial geometry with SIG, categorized by the mean SIG of the BA (Figure 1).

### 2.5 Assessment of carotid stenosis with carotid duplex ultrasonography

CDU was performed using high-frequency linear transducers on LOGIQ E9 (GE, Milwaukee, WI, USA), iU22, or Affiniti 70G (Philips) ultrasound systems. To assess proximal ICA hemodynamic stenosis, parameters measured included peak systolic velocity (PSV), PSV ratio (ICA PSV/CCA PSV), and St. Mary’s ratio (ICA PSV/CCA EDV). Hemodynamic stenosis was classified as: normal (no ICA stenosis or plaque); <50% stenosis (significant plaque with PSV < 125 cm/s, PSV ratio <2, St. Mary’s ratio <8); and ≥50% stenosis (PSV ≥ 125 cm/s, PSV ratio ≥2, or St. Mary’s ratio ≥8). Extracranial ICA stenosis measurement and interpretation were performed by KH with over 20 years of ultrasonography experience.

### 2.6 Statistical analysis

Descriptive data for the clinical characteristics and laboratory findings were presented as means ± standard deviation or percentages, as appropriate. Differences among participants in the three groups were analyzed using one-way analysis of variance with *post-hoc* tests and the chi-squared test. The intraclass coefficient (ICC), along with 95% confidence intervals (CI), was calculated to assess the reliability between measurers. Binary logistic regression with a likelihood ratio test for trend was conducted to assess independent associations between mean SIG and ICA stenosis, adjusting for confounders. Multinomial regression analysis was used to examine factors affecting mean SIG in intracranial arteries, adjusting for confounders including MR acquisition parameters (FA, TR, and TE). Statistical significance was set at *p* < 0.05, and all analyses were performed using IBM SPSS Statistics version 24 (IBM Corp., Armonk, NY, USA). For a determination of sample size, G*Power 3.1 software was used. Assuming a significance level (*α*) of 0.05, an expected effect size (*f*^2^) of 0.15, a power (1 − *β*) of 0.95, and 10 predictors, the analysis indicated that a minimum total sample size of 172 participants was required to achieve adequate statistical power. The statistical analysis was performed by KH and CH.

## 3 Results

**Figure 2:**
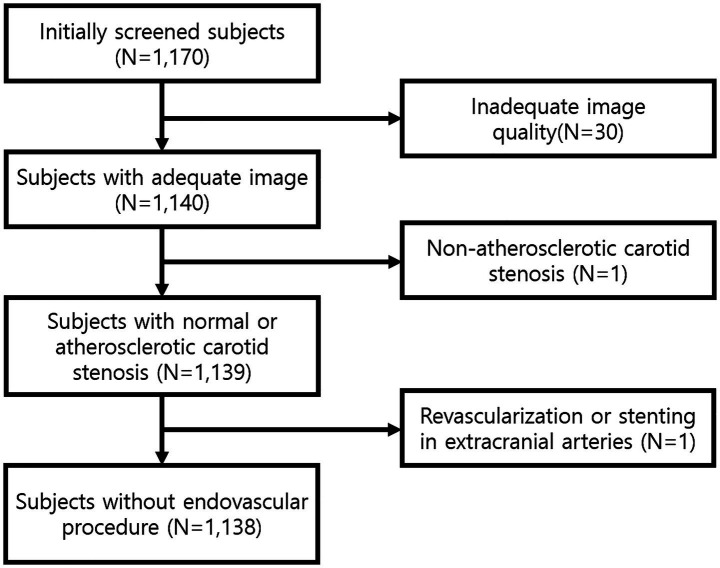
Flow diagram of study population.

From the initial 1,170 screened participants, 30 were excluded due to inadequate intracranial TOF-MRA image quality, one was excluded because of carotid stenosis from non-atherosclerotic causes, and one was excluded due to a history of revascularization or stenting in intra- or extracranial arteries (Figure 2).

A total of 1,138 participants were finally enrolled (mean age ± SD, 63.3 ± 9.6 years, 651 men). Subjects were categorized into three groups based on the severity of ICA stenosis: none, less than 50, and 50% or more (Table 1). For ICA stenosis ≥50%, associations were observed with age, male sex, hypertension, type 2 diabetes, hyperlipidemia, smoking, ICAS, and HbA1c. Among the cerebral arteries, arterial wall SIGs of both the ACAs, PCAs, and the BA showed associations with ICA stenosis, while the ICAs and MCAs did not (Table 2).

**Table 1:** Characteristics according to extracranial ICA status in health screening participants.

Variables	Status of extracranial ICA			*p-*value	*Post-hoc* analysis
	Normal	Stenosis <50%	Stenosis ≥50%		
Number (%)	445 (39.1)	562 (49.4)	131 (11.5)		
Demographics					
Age (years)	59.9 ± 9.9	64.8 ± 8.5	67.5 ± 8.8	<0.001	a < b < c
Male (%)	229 (51.5)	340 (60.5)	82 (62.6)	0.007	
CVD risk factors					
Hypertension	152 (34.5)	280 (50.5)	73 (56.6)	<0.001	
Type 2 diabetes	61 (13.8)	122 (21.9)	33 (25.8)	0.001	
Hyperlipidemia	169 (38.3)	256 (46.1)	64 (50.0)	0.014	
Smoking					
None	262 (59.4)	291 (52.4)	68 (52.7)		
Ex-smoker	106 (24.0)	184 (33.2)	42 (32.6)	0.033	
Current smoker	73 (16.6)	80 (14.4)	19 (14.7)		
Intracranial artery stenosis	32 (7.2)	92 (16.5)	32 (25.2)	<0.001	
Atrial fibrillation	7 (1.6)	19 (3.4)	3 (2.3)	0.191	
Chronic kidney disease	19 (4.3)	25 (4.5)	5 (3.8)	0.947	
Vital signs					
Systolic blood pressure, mmHg	119.9 ± 18.7	122.7 ± 19.3	122.6 ± 20.1	0.079	
Diastolic blood pressure, mmHg	74.2 ± 11.1	73.6 ± 10.8	72.2 ± 10.6	0.222	
Pulse pressure, mmHg	45.7 ± 12.7	49.1 ± 13.7	50.3 ± 16.0	<0.001	a < b,c
Heart rate, rate/min	80.3 ± 15.6	76.2 ± 15.1	74.1 ± 13.6	<0.001	a > b,c
Laboratory					
HbA1c, %	5.9 ± 0.6	6.1 ± 0.8	6.0 ± 0.6	0.003	a < b
hs-CRP, mg/L	0.2 ± 0.6	0.2 ± 0.9	0.1 ± 0.2	0.261	
GFR (mL/min/1.73 m2)	84.5 ± 15.8	84.8 ± 14.8	83.1 ± 13.0	0.501	
Total cholesterol, mg/dL	194.3 ± 41.9	188.6 ± 43.4	186.4 ± 44.4	0.057	
Triglycerides, mg/dL	108.8 ± 61.4	114.5 ± 66.0	101.7 ± 55.8	0.078	
High-density lipoprotein, mg/dL	56.4 ± 16.1	54.5 ± 15.0	56.9 ± 17.2	0.080	
Low-density lipoprotein, mg/dL	118.6 ± 37.2	114.0 ± 37.6	113.0 ± 39.0	0.108	

CVD, cardiovascular disease; GFR, glomerular filtration rate; hs-CRP, high-sensitivity c-reactive protein; ICA, internal carotid artery.

*p*-values are derived from analysis of variance (ANOVA) or chi-square tests, as appropriate, with *post hoc* analysis using Tukey’s HSD test. a–c indicate group categories: a = normal, b = stenosis <50%, c = stenosis ≥50%.

**Table 2:** Mean SIG and blood flow velocity of cerebral arteries according to the status of extracranial ICA.

	Status of extracranial ICA			*p-*value	*Post-hoc* analysis
	Normal	Stenosis <50%	Stenosis ≥50%		
Mean SIG of cerebral arteries, SI/mm					
Distal segment of ICA, Rt	8.66 ± 1.28	8.62 ± 1.35	8.39 ± 1.51	0.130	
Distal segment of ICA, Lt	8.29 ± 1.21	8.31 ± 1.29	8.32 ± 1.46	0.971	
Anterior cerebral artery, Rt	7.76 ± 1.45	7.58 ± 1.43	7.36 ± 1.59	0.016	a > c
Anterior cerebral artery, Lt	7.80 ± 1.25	7.69 ± 1.32	7.34 ± 1.61	0.003	a,b > c
Middle cerebral artery, Rt	7.72 ± 1.32	7.65 ± 1.31	7.54 ± 1.56	0.389	
Middle cerebral artery, Lt	7.63 ± 1.24	7.52 ± 1.23	7.33 ± 1.46	0.056	a > c
Posterior cerebral artery, Rt	8.92 ± 1.85	8.69 ± 1.84	8.17 ± 1.70	<0.001	a,b > c
Posterior cerebral artery, Lt	9.08 ± 1.84	8.64 ± 1.86	8.29 ± 1.59	<0.001	a > b,c
Basilar artery (BA)	10.00 ± 1.67	9.59 ± 1.63	9.03 ± 1.40	<0.001	a > b > c
PSV of extracranial arteries, cm/s					
CCA, Rt	74.8 ± 19.4	72.1 ± 35.9	73.1 ± 20.6	0.346	
CCA, Lt	78.8 ± 24.9	74.8 ± 21.4	75.1 ± 23.1	0.022	a > b
ICA, Rt	66.5 ± 19.4	64.9 ± 19.0	72.0 ± 23.0	0.001	c > a,b
ICA, Lt	65.9 ± 18.1	66.9 ± 21.4	70.5 ± 22.7	0.078	
EDV of extracranial arteries, cm/s					
CCA, Rt	22.5 ± 7.2	21.1 ± 12.5	20.8 ± 7.5	0.060	
CCA, Lt	24.6 ± 17.8	22.1 ± 10.4	20.9 ± 8.6	0.003	a > b,c
ICA, Rt	24.1 ± 9.5	24.0 ± 33.9	22.0 ± 9.1	0.659	
ICA, Lt	24.3 ± 9.8	23.5 ± 10.3	22.8 ± 8.7	0.202	

SI, signal intensity; EDV, end diastolic velocity; PSV, peak systolic velocity; SIG, signal intensity gradient, ICA, internal carotid artery, CCA, common carotid artery.

*p*-values are derived from analysis of variance (ANOVA) or chi-square tests, as appropriate, with *post hoc* analysis using Tukey’s HSD test. a–c indicate group categories: a = normal, b = stenosis <50%, c = stenosis ≥50%.

To analyze the association between the mean SIG of the BA and ICA stenosis ≥50%, univariate and multivariate logistic regression analyses were performed (Table 3). Multivariate logistic regression analysis showed that both age and mean SIG of the BA were independently associated with ICA stenosis, even after adjusting for potential confounders, including sex, conventional risk factors, and ICAS. Compared to the group in the highest tertile of BA SIG, the second tertile had an adjusted odds ratio (aOR) of 3.20 (95% CI: 1.70–6.04), while the lowest tertile had an aOR of 3.68 (95% CI: 1.97–6.88). The likelihood ratio test for trend among the three BA SIG groups was *p* < 0.001.

**Table 3:** Logistic regression analyses for moderate to severe stenosis of ICA in health screening participants.

Variables	ICA stenosis ≥50%			
	Crude OR (95% CI)	*p-*value	Adjusted OR* (95% CI)	*p-*value
Age	1.06 (1.04–1.08)	<0.001	1.05 (1.02–1.08)	<0.001
Sex	0.79 (0.55–1.14)	0.214		
Smoking	1.11 (0.77–1.61)	0.564		
Hypertension	1.70 (1.18–2.43)	0.004		
Type 2 diabetes	1.49 (0.98–2.27)	0.061		
Hyperlipidemia	1.41 (0.98–2.02)	0.062		
Intracranial artery stenosis	2.32 (1.50–3.58)	<0.001		
Systolic blood pressure	1.00 (0.99–1.01)	0.527		
Heart rate	0.98 (0.97–0.99)	0.005		
HbA1c	1.08 (0.99–1.00)	0.498		
Total cholesterol	1.00 (0.99–1.00)	0.241		
Mean SIG value of BA				
3rd tertile (highest mean SIG)	1.00		1.00	
2nd tertile (middle mean SIG)	3.93 (2.21–6.99)	<0.001	3.20 (1.70–6.04)	<0.001
1st tertile (lowest mean SIG)	4.24 (2.39–7.51)^†^	<0.001	3.68 (1.97–6.88)^‡^	<0.001

* Adjusted by: age, sex, smoking, hypertension, diabetes mellitus, hyperlipidemia, intracranial artery stenosis, systolic blood pressure, heart rate, HbA1c, total cholesterol, and mean SIG of BA.

^†^*p*-value <0.001, ^‡^*p* = 0.001 by likelihood ratio test for trend.

BA, basilar artery; CI, confidence intervals; OR, odds ratio; SIG, signal intensity gradient.

Given that the mean SIG values of the BA demonstrated the strongest association with ICA stenosis, individuals were categorized into three groups based on the tertiles of mean BA SIG (Supplementary Table 1). The group in the lowest tertile of BA SIG was the oldest, had the highest proportion of male participants, and exhibited the longest MR acquisition parameters –including FA, TR, and TE—among the three groups (*p* < 0.001). Hypertension, type 2 diabetes, hyperlipidemia, atrial fibrillation, and ICAS were most prevalent in the lowest tertile group (all *p* values < 0.05).

Extracranial ICA stenosis ≥50% was observed in 15.6% of individuals in the lowest tertile of BA SIG, compared to 4.2% in the highest tertile (*p* < 0.001, Supplementary Table 2). In terms of CDU parameters, the group in the lowest tertile of BA SIG had the lowest PSV and EDV in the carotid segments, with all *p* values < 0.05 except for the right ICA PSV. There was no difference in CCA IMTs. The mean SIGs of other cerebral arteries were also lowest in the group with the lowest tertile of BA SIG (all *p* values < 0.001).

Multinomial logistic regression analysis was performed to identify factors associated with the mean SIG values of the BA (Table 4). Compared to the group with the highest BA SIG, the group with the lowest BA SIG had a higher aOR (95% CI) for ICA stenosis: 2.72 (1.27–5.82, *p* = 0.010) and higher aORs for hypertension and lower aOR for left CCA mean diastolic velocity: 1.61 (1.05–2.46, *p* = 0.028) and 0.95 (0.93–0.98, *p* = 0.001), respectively. The second tertile group showed similar findings. All three MR parameters demonstrated significant associations with BA SIG values. Notably, even adjusting for these parameters, the significant association between ICA stenosis and BA SIG remained robust.

**Table 4:** Multinomial logistic regression analysis for the tertiles of the mean SIG of the BA.

Mean SIG value of BA	Variables	Adjusted OR (95% CI)*	*p-*value
3rd tertile (highest mean SIG)		Reference	
2nd tertile (middle mean SIG)	Flip angle (FA)	0.60 (0.40–0.91)	0.017
	Repetition time (TR)	1.50 (1.23–1.84)	<0.001
	Echo time (TE)	2.00 (1.20–3.33)	0.008
	ICA stenosis (≥50%)	2.89 (1.41–5.93)	0.004
1st tertile (lowest mean SIG)	FA	0.47 (0.30–0.73)	0.001
	TR	1.57 (1.26–1.94)	<0.001
	TE	3.17 (1.90–5.30)	<0.001
	Sex	0.63 (0.42–0.96)	0.032
	Hypertension	1.61 (1.05–2.46)	0.028
	Lt CCA MDV	0.95 (0.93–0.98)	0.001
	ICA stenosis (≥50%)	2.72 (1.27–5.82)	0.010

* Adjusted by: age, sex, heart rate, total cholesterol, mean-diastolic velocity (MDV) of left CCA, hypertension, diabetes mellitus, hyperlipidemia, intracranial artery stenosis, FA, TR, TE, and stenosis of internal carotid artery.

BA, basilar artery; CCA MDV, mean diastolic velocity of common carotid artery.

To assess measurement reliability, the mean SIG values were repeatedly evaluated in 10 subjects by two independent observers. The results demonstrated excellent reproducibility, with high ICCs for both interobserver and intraobserver agreement: 0.96 (95% CI, 0.91–0.98, *p* < 0.001) and 0.91 (0.76–0.99, *p* < 0.001), respectively.

## 4 Discussion

In this research, we reveal that extracranial ICA stenosis was associated with the hemodynamic parameter of cerebral arteries, the SIG derived from TOF-MRA. The low BA SIG demonstrated the strongest association with ICA stenosis of all the cerebral arteries (OR: 3.68, 95% CI: 1.97–6.88; *p* < 0.001). Additionally, low BA SIG was associated with vascular risk factors, including hypertension, type 2 diabetes, hyperlipidemia, and atrial fibrillation.

Extracranial ICA stenosis is a key cause of ischemic stroke and has been extensively studied for its local geometric and hemodynamic disturbances, including characteristic branching with bulb formation and distinct zones of high and low wall shear stress (16, 17). In this study, significant stenosis (≥50%) was defined using carotid ultrasonography. In subjects with atherosclerotic stenosis, the hemodynamic features may evolve differently from those of the original geometry (18), and carotid stenosis has been reported to interact with intracranial cerebral hemodynamics (19). The lack of a significant association between the SIG values of the distal ICA and proximal extracranial ICA stenosis in this study suggests that the hemodynamic features of the distal ICA may be modified by the proximal carotid status.

In cases of extracranial ICA stenosis, when the flow in the ICA territory is compromised, the cerebral arteries in the circle of Willis should be carefully examined for potential compensatory changes from primary or secondary collaterals (20). The significant and independent negative association between BA SIG and ICA stenosis in this study suggests two possible explanations: First, stenosis-affected flow may not be sufficiently reduced to trigger collateral formation. Second, other cerebral arteries—aside from the stenosed ICA—may also experience similarly impaired flow conditions in subjects within the lowest tertile of BA SIG. The second hypothesis suggests that vascular pathology may affect not only cerebral arteries but also systemic circulation. Given that the vascular system originates from the heart and extends throughout the body, individual vessels do not function in isolation. The documented association between coronary and carotid artery stenosis supports the presence of pathological changes across different components of the vascular network (21).

Unhealthy flow conditions in the extra- and intracranial arteries may accelerate the atherosclerotic process, particularly in regions with atheroprone geometric features, such as the ICA bulb. A similar phenomenon may occur in the intracranial cerebral arteries, particularly in regions of branching or angulation, leading to ICAS. In this study, ICAS was significantly associated with BA SIG. The interactive effects of flow conditions and geometric features are well-documented (22), but further studies are warranted in the field of CVD due to the limited availability of hemodynamic information in cerebral arteries.

In the subjects with extracranial ICA stenosis, even if asymptomatic, blood flow to the distal segment of ICA and MCA might be compromised. Compared to the arterial beds of the anterior circulation, the BA, part of the posterior circulation, follows a relatively straight trajectory (23), and its flow remains uncompromised. In addition, the BA possesses anatomically unique characteristics that make it particularly suited for examination with TOF-MRA. Its alignment along the z-axis renders it perpendicular to the imaging plane in TOF-MRA, resulting in optimal arterial signal intensity. The BA is richly innervated by neuronal fibers of parasympathetic origin, providing a powerful vasodilatory mechanism (24). However, the BA is susceptible to various pathologies, including angulation (25), dolichoectasia (26), fenestration (27), as well as atherosclerotic stenosis (28). Further studies are needed to determine whether the BA and its hemodynamics could serve as a marker for cerebrovascular flow status.

In this study, BA SIG was positively associated with heart rate and flow velocities in the extracranial carotid arteries. Although the positive association between BA SIG and carotid flow velocities, even among individuals with critical stenosis, may seem counterintuitive, it could be attributed to an averaging effect that offsets the influence of the 15.8% of subjects with critical stenosis, consistent with previous findings (29). Cardiac output, determined by stroke volume and heart rate, has an independent effect on cerebral blood flow. The positive associations between BA SIG, heart rate, and carotid flow velocities suggest the presence of a common underlying pathophysiological mechanism. This study could not determine whether the observed phenomenon was due to comorbid vascular risk factors or a cardiovagal neural effect (30).

The SIG from TOF-MRA is calculated along arterial walls, using an analogy to shear rate (du/dr), where du is replaced by the difference in signal intensities. The primary feature of SIG is its combination of geometric and *in-vivo* hemodynamic information obtained directly from TOF-MRA. This study extends previous research by demonstrating significant associations between arterial SIG values and ICA stenosis or vascular risk factors.

This study has limitations. First, it was a single-center, single-ethnicity, retrospective study, stressing that further studies across multiple centers and diverse ethnic groups are essential to ensure the generalizability of the results. Second, the study relies on a single TOF-MRA examination, which does not account for inter-examination variability. Even in the same subject, different SIG values may be observed in repeat examinations. Third, the degree of ICA stenosis was determined using CDU, which allows real-time blood flow velocity measurement but limits arterial wall assessment. Further studies are required to clarify the direct relationship between carotid and cerebral arterial hemodynamic features. Fourth, given the cross-sectional design of this study, it was not possible to establish a causal relationship between ICA stenosis and BA SIG. To elucidate the nature and direction of this association, future research employing a prospective longitudinal design is warranted. Last, it is not a randomized study. The present study targeted three groups clearly defined based on specific criteria; therefore, randomization was not deemed necessary.

## 5 Conclusion

This study demonstrated that extracranial ICA stenosis was significantly associated with BA SIG derived from TOF-MRA, even after adjusting for potential confounders. The cerebral artery SIG measured by TOF-MRA may serve as a useful marker for predicting atherosclerotic burden and potential risk for brain ischemia. To elucidate the causal relationship between ICA stenosis and BA SIG, a prospective longitudinal study involving multiple centers and diverse ethnic populations is warranted.

## Data Availability

The raw data supporting the conclusions of this article will be made available by the authors, without undue reservation.
