# Supplementary material for: Association of carotid artery stenosis with cerebral artery signal intensity gradient on time-of-flight magnetic resonance angiography
Source: Front Neurol. 2025 Aug 11;16:1576655. doi: 10.3389/fneur.2025.1576655 (PMC12375817; doi:10.3389/fneur.2025.1576655)
Supplement: Supplementary file 1 [file Table_1.docx]

**Supplementary Materials**

**Supplementary figure.** A representative image for a measurement of SIG values in intracranial cerebral arteries, as captured from the NeuFlow^TM^.


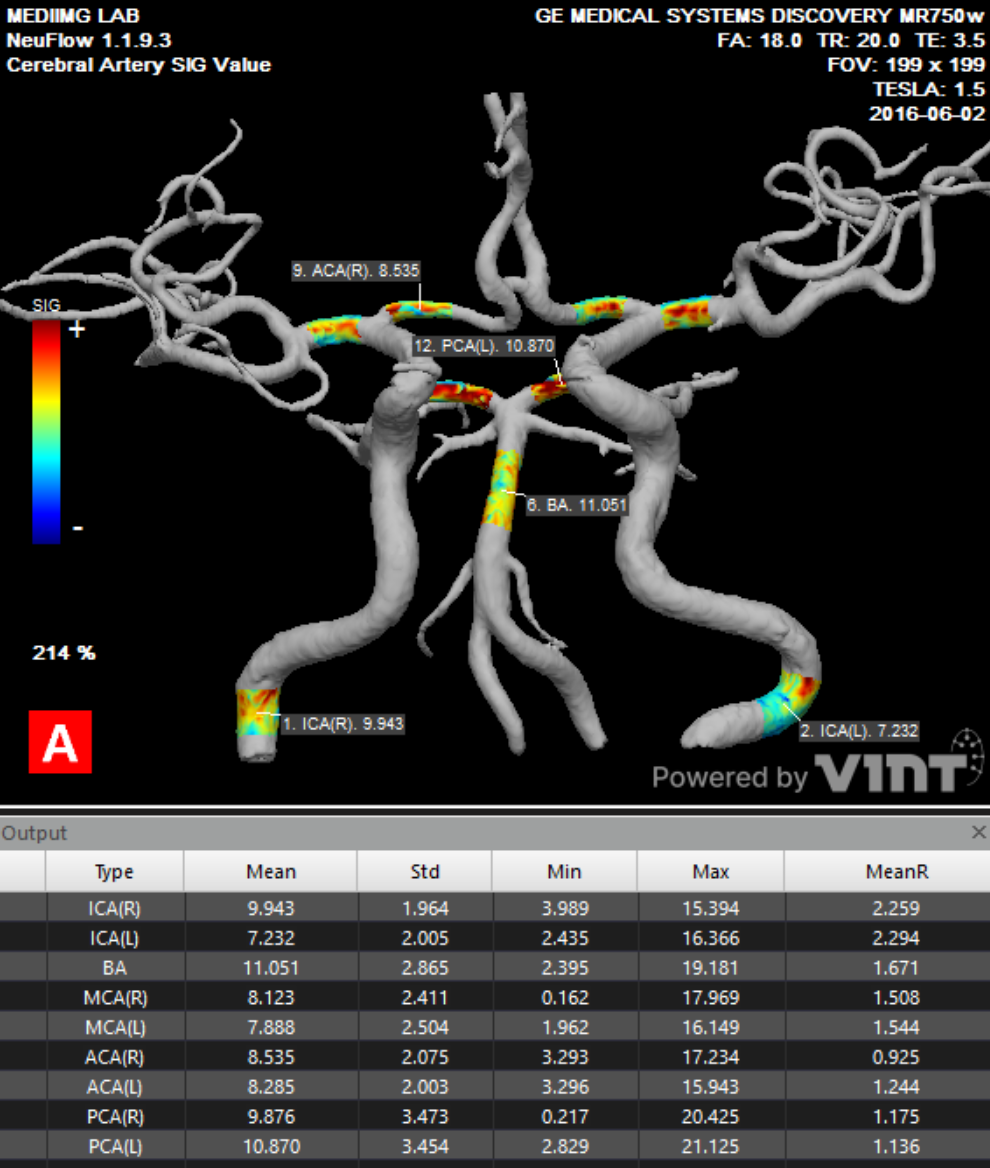


| **Supplementary table 1. Baseline characteristics according to the mean values of SIG in basilar artery (BA) among healthy subjects** | | | | | |
| --- | --- | --- | --- | --- | --- |
| Variables | Mean SIG value of BA | | | *p* value |  |
|  | 1^st^ tertile | 2^nd^ tertile | 3^rd^ tertile |  |  |
| Number (%) | 379 (33.3) | 380 (33.4) | 379 (33.3) |  |  |
| **mean SIG of BA, SI/mm, range** | 3.50-8.95 | 8.95-10.42 | 10.42-14.82 |  |  |
| **Demographics** |  |  |  |  |  |
| Age (years) | 65.4±9.3 | 63.2±9.7 | 61.0±9.0 | <0.001 | a>b>c |
| Male (%) | 235 (62.0) | 215 (56.6) | 200 (52.8) | 0.022 |  |
| **CVD risk factors** |  |  |  |  |  |
| Hypertension | 204 (53.8) | 168 (44.2) | 132 (34.8) | <0.001 |  |
| Diabetes mellitus | 89 (23.5) | 66 (17.4) | 59 (15.6) | 0.013 |  |
| Hyperlipidemia | 188 (49.6) | 174 (45.8) | 126 (33.2) | <0.001 |  |
| Smoking |  |  |  |  |  |
| None | 203 (53.6) | 204 (53.7) | 210 (55.4) |  |  |
| Ex-smoker | 112 (29.6) | 118 (31.1) | 110 (29.0) | 0.948 |  |
| Current smoker | 64 (16.9) | 58 (15.3) | 59 (15.6) |  |  |
| Intracranial artery stenosis | 63 (16.6) | 56 (38.0) | 36 (9.5) | 0.008 |  |
| Atrial fibrillation | 17 (4.5) | 7 (1.8) | 5 (1.3) | 0.011 |  |
| Chronic kidney disease | 18 (4.7) | 16 (4.2) | 14 (3.7) | 0.748 |  |
| **Vital signs** |  |  |  |  |  |
| Systolic blood pressure, mmHg | 122.9±20.4 | 118.8±18.8 | 123.4±18.2 | 0.002 | b<c |
| Diastolic blood pressure, mmHg | 74.8±11.1 | 71.7±9.9 | 74.8±11.4 | <0.001 | b<a,c |
| Pulse pressure, mmHg | 48.2±14.5 | 47.1±13.9 | 48.6±12.7 | 0.346 |  |
| Heart rate, /min | 74.9±13.1 | 76.9±14.9 | 81.0±17.0 | <0.001 | a,b<c |
| **Laboratory** |  |  |  |  |  |
| HbA1c, % | 6.0±0.8 | 6.0±0.7 | 5.9±0.7 | 0.080 |  |
| hs-CRP, mg/L | 0.2±0.6 | 0.1±0.3 | 0.2±1.0 | 0.164 |  |
| GFR (mL/min/1.73m^2^) | 84.0±15.8 | 84.6±14.9 | 85.0±14.3 | 0.687 |  |
| Total cholesterol, mg/dL | 185.4±44.1 | 188.6±40.6 | 197.7±43.5 | <0.001 | a,b<c |
| Triglycerides, mg/dL | 121.0±74.4 | 105.2±56.7 | 106.2±56.2 | 0.001 | a>b,c |
| HDL cholesterol, mg/dL | 52.8±13.6 | 55.7±15.1 | 58.0±17.8 | <0.001 | a<b,c |
| LDL cholesterol, mg/dL | 113.0±37.6 | 115.0±35.7 | 119.0±39.4 | 0.084 |  |
| **MR parameters** |  |  |  |  |  |
| Flip angle | 19.8±2.2 | 19.3±2.1 | 18.1±0.5 | <0.001 | a>b>c |
| Repetition time | 24.0±4.1 | 22.7±4.1 | 20.1±0.9 | <0.001 | a>b>c |
| Echo time | 5.3±1.7 | 4.6±1.6 | 3.6±0.5 | <0.001 | a>b>c |
| Mean ± standard deviation (SD) or number (%) shown as appropriate. SIG=signal intensity gradient; BA=basilar artery; CVD=cardiovascular disease; Hs-CRP=high-sensitivity c-reactive protein; GFR=glomerular filtration rate; HDL=high density lipoprotein; LDL=low density lipoprotein  *p* values by analysis of variance (ANOVA) or chi-square test as appropriate, and post hoc analysis by Tukey's HSD test | | | | | |

| **Supplementary Table 2. Hemodynamic characteristics according to the mean values of SIG in BA** | | | | | |
| --- | --- | --- | --- | --- | --- |
| Variables | Mean SIG value of BA | | | *p* value |  |
|  | 1^st^ tertile | 2^nd^ tertile | 3^rd^ tertile |  |  |
| **ICA status** |  |  |  |  |  |
| Normal | 115 (30.3) | 147 (38.7) | 186 (49.1) |  |  |
| Mild stenosis (<50%) | 203 (53.6) | 178 (46.8) | 178 (47.0) | <0.001 |  |
| Moderate to severe stenosis (≥50%) | 59 (15.6) | 56 (14.7) | 16 (4.2) |  |  |
| **Findings of carotid sonography** |  |  |  |  |  |
| Average CCA IMT, Rt, mm | 0.6±0.1 | 0.7±0.2 | 0.6±0.1 | 0.396 |  |
| Average CCA IMT, Lt, mm | 0.7±0.2 | 0.7±0.2 | 0.6±0.2 | 0.267 |  |
| PSV of CCA, Rt, cm/sec | 69.7±18.7 | 73.4±20.5 | 76.2±40.9 | 0.008 | a<c |
| PSV of CCA, Lt, cm/sec | 73.4±21.7 | 76.6±22.0 | 78.5±20.8 | 0.006 | a<c |
| PSV of ICA, Rt, cm/sec | 64.7±19.6 | 69.0±28.9 | 68.4±44.4 | 0.165 |  |
| PSV of ICA, Lt, cm/sec | 63.9±19.7 | 68.3±20.6 | 68.3±20.7 | 0.004 | a<b,c |
| EDV of CCA, Rt, cm/sec | 19.8±8.6 | 22.2±13.5 | 22.7±7.1 | <0.001 | a<c |
| EDV of CCA, Lt, cm/sec | 21.0±11.6 | 22.7±8.3 | 25.0±18.5 | <0.001 | a<b,c |
| EDV of ICA, Rt, cm/sec | 21.6±8.5 | 23.6±11.0 | 26.5±41.3 | 0.028 | a<c |
| EDV of ICA, Lt, cm/sec | 21.7±8.5 | 24.3±10.8 | 24.9±10.2 | <0.001 | a<b,c |
| **Mean SIG of cerebral arteries, SI/mm** | |  |  |  |  |
| Distal segment of ICA, Rt | 7.73±1.40 | 8.79±0.99 | 9.29±1.10 | <0.001 | a<b<c |
| Distal segment of ICA, Lt | 7.62±1.41 | 8.56±1.05 | 8.73±1.05 | <0.001 | a<b,c |
| Anterior cerebral artery, Rt | 6.59±1.57 | 7.76±1.14 | 8.48±0.91 | <0.001 | a<b<c |
| Anterior cerebral artery, Lt | 6.73±1.49 | 7.90±1.04 | 8.42±0.76 | <0.001 | a<b<c |
| Middle cerebral artery, Rt | 6.82±1.55 | 7.93±1.00 | 8.22±0.97 | <0.001 | a<b<c |
| Middle cerebral artery, Lt | 6.72±1.37 | 7.73±0.98 | 8.16±0.92 | <0.001 | a<b<c |
| Posterior cerebral artery, Rt | 7.39±1.67 | 8.74±1.47 | 10.02±1.33 | <0.001 | a<b<c |
| Posterior cerebral artery, Lt | 7.45±1.56 | 8.68±1.42 | 10.18±1.41 | <0.001 | a<b<c |
| CCA=common carotid artery; IMT=intima-media thickness; Rt=right; Lt=left; PSV=peak systolic velocity; EDV=end diastolic velocity.  *p* values by ANOVA or chi-square test as appropriate, and post hoc analysis by Tukey's HSD test | | | | | |
